# Titanium Dioxide Nanoparticles Negatively Influence Gill Metabolism in *Pinctada fucata martensii*

**DOI:** 10.3390/metabo14120682

**Published:** 2024-12-05

**Authors:** Heqi Zou, Fengfeng Li, Luomin Huang, Jiaying Yao, Yujing Lin, Chuangye Yang, Ruijuan Hao, Robert Mkuye, Yongshan Liao, Yuewen Deng

**Affiliations:** 1 Fisheries College, Guangdong Ocean University, Zhanjiang 524088, China; 11135232mm@stu.gdou.edu.cn (H.Z.); 2112201126@stu.gdou.edu.cn (F.L.); huangluomin@stu.gdou.edu.cn (L.H.); 11135128zz@stu.gdou.edu.cn (J.Y.); 11135419rr@stu.gdou.edu.cn (Y.L.); yangcy@gdou.edu.cn (C.Y.); 1252101432@stu.gdou.edu.cn (R.M.); dengyw@gdou.edu.cn (Y.D.); 2 Guangdong Science and Innovation Center for Pearl Culture, Zhanjiang 524088, China; 3 Pearl Breeding and Processing Engineering Technology Research Centre of Guangdong Province, Zhanjiang 524088, China; 4 Guangdong Provincial Key Laboratory of Aquatic Animal Disease Control and Healthy Culture, Zhanjiang 524088, China; 5 Development and Research Center for Biological Marine Resources, Southern Marine Science and Engineering Guangdong Laboratory (Zhanjiang), Zhanjiang 524088, China; haorj@zjblab.com; 6 Pearl Research Institute, Guangdong Ocean University, Zhanjiang 524088, China

**Keywords:** *Pinctada fucata martensii*, titanium dioxide nanoparticles, energy metabolism, metabolomics

## Abstract

Background: In recent years, titanium dioxide (TiO_2_) nanoparticles (NPs) have been widely used in various industries due to their favorable chemical properties, and their contamination of the environment has attracted much attention, especially to aquatic animals. Methods: Therefore, we assessed the impact of TiO_2_ NPs (5 mg/L) on the marine bivalve, pearl oyster (*Pinctada fucata martensii*), especially gill metabolism. Pearl oysters were exposed to seawater containing 5 mg/L TiO_2_ NPs for 14 days, followed by 7 days of recovery in untreated seawater. Gill tissues and hepatopancreatic tissues were sampled on days 0, 14, and 21 of the experiment named C0, E14, and R7, respectively. Results: Metabolomic analysis identified 102 significantly different metabolites (SDMs) on gills tissue in pearl oysters following exposure to TiO_2_ NPs (C0 vs. E14). Compared with group C0, group E14 had 76 SDMs (such as acetylcholine, itaconic acid, citric acid, and taurine) with higher concentrations and 26 (including L-arginine and isobutyryl-L-carnitine) with lower concentrations. KEGG (Kyoto Encyclopedia of Genes and Genomes) analysis showed that these SDMs enriched 28 pathways, including glycine, serine, and threonine metabolism, neuroactive ligand–receptor interaction, and taurine and hypotaurine metabolism. In addition, 116 SDMs were identified in E14 and R7 pearl oysters. Compared with group E14, group R7 had 74 metabolites (such as acetylcholine, 6-phosphogluconic acid, isocitric acid, and itaconic acid) with higher concentrations and 42 (including uracil, glycerophosphocholine, N-Acetyl-D-glucosamine) with lower concentrations. The SDMs identified between E14 and R7 enriched 25 pathways, including the pentose phosphate pathway, glutathione metabolism, and citrate cycle (TCA cycle). In addition, analysis of the energy metabolism-associated enzymes revealed that exposure to TiO_2_ NPs reduced Ca^2+^/Mg^2+^-ATPase, Na^+^/K^+^-ATPase, and Total-ATPase activities. Conclusions: These findings suggested that TiO_2_ NPs may inhibit the energy metabolism function of gill and hepatopancreas of pearl oysters. Meanwhile, TiO_2_ NPs may affect the normal functioning of immune and osmoregulatory functions of pearl oysters gill and even may lead to oxidative stress and neurotoxicity. Therefore, this study may provide a reference for analyzing the bioadaptation of marine bivalves to TiO_2_ NPs and the potential negative effects of TiO_2_ NPs on bivalves.

## 1. Introduction

Engineered nanostructures (ENs) ranging from 1 to 100 nanometers in size have emerged as a prominent area for scientific inquiry in the modern era [1]. Among the various ENs, titanium dioxide (TiO_2_) nanoparticles (NPs) are produced worldwide and utilized in multiple commercial products, including paints, construction materials, personal care items, sunscreens, electronics, and food products [2,3]. These particles are unavoidably released into various ecological systems, including the aquatic environment [1]. Consequently, a gradual rise in their levels has been detected in the environment, including up to 1.65 mg/L increase in a few places [4,5]. More importantly, it is predicted that a certain amount of TiO_2_ NPs will enter the food chain and accumulate in species of different organisms [6]. Studies have suggested that the sessile bivalves are the aquatic species most vulnerable to nanoparticle toxicity, probably due to the filter-feeding strategy and sessile lifestyle [7,8,9,10]. Specifically, the agglomerated state NPs are retained in the gills and eventually make their way to the digestive system via endocytosis, resulting in detrimental effects [11,12]. TiO_2_ NPs induce various immune responses in *Ruditapes decussatus* [13] and *Mytilus coruscus* [14]. They cause oxidative stress and are neurotoxic to *Tegillarca granosa* [15,16]. TiO_2_ NPs also lead to delayed embryonic development of *M. galloprovincialis* [9], inhibit the metabolism of *M. coruscus,* and cause DNA damage [17].

Typically, metabolomics, which explores and quantifies intracellular metabolites and their intermediates within cells, tissues, and other biological matrices or small organisms, demonstrates the dynamic reactions of species to genetic modifications and physiological and developmental stimuli [18]. This approach offers vital data concerning the overall shifts in the metabolites of specific biochemical pathways in response to environmental stress or toxic substances [19,20,21]. Thus, metabolomics helps evaluate environmental risks [3,22]. In addition, examining the metabolites of living organisms offers impartial insights into the changes in metabolic pathways during or after exposure to harmful substances. Understanding these changes helps elucidate the mechanism via which the toxin operates within the organism [23]. Metabolomics has proven effective in elucidating the mechanisms underlying TiO_2_ NPs toxicity in aquatic organisms. Raja et al. [24] used metabolomics to find that TiO_2_ NPs disrupted purine, glycerophospholipid, and energy metabolism in zebrafish (*Danio rerio*). Recently, Duan et al. [25] discovered that the exposure of hybrid grouper juveniles to 1–10 mg/L TiO_2_ NPs for 14 days modulated lipid and amino acid metabolism in the liver. Thus, research has proven that metabolomics serves as a potent tool for comprehensively analyzing the negative impact of TiO_2_ NPs in various organisms [24,25].

The pearl oyster (*Pinctada fucata martensii*) is naturally found in the equatorial zone between the Tropic of Cancer and the Tropic of Capricorn in the Indo-Pacific and Western Atlantic regions, such as the coastal areas of South China, Japan, Australia, and Southeast Asia [26,27]. This shellfish has significant economic value due to its capacity to generate premium-quality pearls [3,28,29]. Meanwhile, pile and raft cultures are adopted for culturing [30]. However, pearl oysters cultured via these methods are vulnerable to external elements, such as salinity, temperature fluctuations, and pollution [30,31]. Specific studies have also detected higher concentrations of NPs in the intertidal regions than in other areas [32,33]. Therefore, pearl oysters face a huge threat from TiO_2_ NPs [34,35]. It has been shown that TiO_2_ NPs adversely affect immunity, oxidative stress, and intestinal flora balance in pearl oysters [34,35], but the metabolic mechanisms underlying the response of pearl oysters to them remain unclear. Therefore, the present study utilized non-targeted liquid chromatography–mass spectrometry (LC–MS) to examine the metabolite variations induced by TiO_2_ NPs in the gill tissue of *P. f. martensii* to understand the mechanism of metabolic effects of TiO_2_ NPs on pearl oysters. Thus, the study will serve as a reference for analyzing the impact of TiO_2_ NPs on marine bivalve metabolism.

## 2. Materials and Methods

### 2.1. Exposure of Pearl Oysters to TiO_2_ NPs

Anatase form of TiO_2_ NPs of approximately 5–10 nm in size was purchased from Shanghai McLean Biochemical Technology (Shanghai, China). TiO_2_ NPs were first added to filtered seawater and homogenized for 20 min with an LC-JY92-TTN ultrasonic homogenizer (65W; Lichen Bonsi Instrument Technology, Shanghai, China) to prepare a 1 g/L nanoparticle suspension. The 1 g/L suspension obtained was further diluted with seawater to obtain 5 mg/L TiO_2_ NPs for the experiment.

Pearl oysters were collected from Leizhou Daqiuzhuang Aquatic Products Breeding (Leizhou, China). They were of the black-colored selected line breed [36], and their average shell height was 78.06 ± 3.90 mm. The pearl oysters with intact and uniform-sized shells were randomly selected and cultured in cylindrical barrels (300 L) for 7 days after removing the adherents on the shell surface. The cylindrical barrels contain three replicates of thirty pearl oysters each.

These pearl oysters were exposed to 5 mg/L TiO_2_ NPs for 14 days (treatment phase) and further cultured in seawater without TiO_2_ NPs for 7 days (recovery phase). Throughout this period, the water in the barrel was changed every day, and pearl oysters were fed unicellular algae (20,000 cells/mL *Chlorella* and 10,000 cells/mL *Platymonas subcordiformis*). Following the 1 h of feeding, TiO_2_ NPs solution was added to each tank according to the treatment group to maintain the concentration of 5 mg/L TiO_2_ NPs. On days 0 (control C0), 14 (E14), and 21 (recovered, R7), three pearl oysters were randomly sampled from each replicate. Then, the gill and hepatopancreatic tissues were sampled, instantly frozen in liquid nitrogen, and kept at −80 °C for further analysis.

### 2.2. Analysis of Various Biochemical Parameters

Hepatopancreatic tissues were accurately weighed and mechanically homogenized by adding 9 times the volume of saline at the ratio of weight (g): volume (mL) = 1:9 under ice water bath conditions. The extract was centrifuged at 12,000× *g* at 4 °C for 20 min, and the supernatant was transferred into a 2 mL tube. Further, the content of protein in the supernatant was measured following the Lowry method; here, bovine serum albumin was used as the standard. Meanwhile, Ca^2+^/Mg^2+^-ATPase (CMA), total-ATPase (T-ATP) and Na^+^/K^+^-ATPase (NKA) activities were assayed according to the kits purchased from Nanjing Jianjian Institute of Bioengineering. Their absorbance (636 nm) was assessed using an automated microplate reader (EnSpire, PerkinElmer, USA), and enzyme activities were calculated according to the manufacturer’s instructions.

### 2.3. Extraction and Identification of Metabolites

The gill tissues (25 ± 1 mg) were mixed with beads and extraction solution [500 μL of a mixture of methanol, acetonitrile, and water, 2:2:1 (*v*/*v*)], containing deuterated internal standards, vortexed for about 30 s, homogenized (4 min, 35 Hz), and again sonicated for 5 min in a cold water bath (4 °C); this step was carried out thrice. Then, the sample was further incubated in a freezer (−40 °C) for 1 h and centrifuged for 15 min at 4 °C and 12,000 rpm (R = 8.6 cm; RCF = 13,800× *g*) to obtain the supernatant, and the supernatant was transferred to a fresh glass vial for Liquid chromatography with tandem mass spectrometry (LC-MS/MS) analysis. All supernatant samples were mixed in equal volumes to obtain the quality control (QC) sample.

### 2.4. Liquid Chromatography-Tandem Mass Spectrometry

LC–MS/MS was carried out on a Vanquish UHPLC system (Thermo Fisher Scientific, Waltham, MA, USA) with an ACQUITY UPLC BEH Amide (Waters; 1.7 μm, 2.1 mm × 50 mm) coupled to an Orbitrap Exploris 120 mass spectrometer (Orbitrap MS; Thermo Scientific, Waltham, MA, USA) to analyze the metabolites in the sample extracts. A combination of ammonia hydroxide (25 mmol/L) and ammonium acetate (25 mmol/L) in water (A; pH = 9.75) and acetonitrile (B) were used as the mobile phases. The injection volume in the auto-sampler, operating at 4 °C, was set at 2 µL. Finally, the Orbitrap Exploris 120 mass spectrometer, controlled by an acquisition software 4.4 (Xcalibur, Thermo), acquired the MS/MS spectra of the samples in an information-dependent acquisition (IDA) mode. In this mode, the acquisition software continuously evaluates the full scan MS spectrum. The mass spectrometer was operated in the electron spray ionization (ESI) mode with the various features set as follows: 50 Arb, sheath gas flow rate; 15 Arb, Aux gas flow rate; 320 °C, capillary temperature; 60,000, full MS resolution; 15,000 MS/MS resolution; 20/30/40, collision energy range. The spray voltage for positive or negative mode was adjusted to 3.8 kV or −3.4 kV, respectively.

### 2.5. Analysis of Experimental Data

#### 2.5.1. Analysis of Metabolomic Data

The ProteoWizard software 3.0.22317 converted the original MS data into the mzXML format, which was further analyzed in R utilizing XCMS for peak identification, retrieval, alignment, and fusion. Subsequently, the MS2 database (Biotree DB) developed in-house was utilized for metabolite identification. Further, the cutoff for annotation was set at 0.3. For the final data set, including sample names, peak counts, and normalized peak areas, SIMCA16.0.2 (Sartorius Stedim Data Analytics AB, Sweden) was employed to conduct multivariate analysis. Following the methodology proposed by Yang et al. [21], principal component analysis (PCA) was performed to assess the grouping and scattering of the samples, and orthogonal projections to latent structures-discriminate analysis (OPLS-DA) was carried out to generate a predictive model and assess the metabolite differences among the various samples. The variable importance in the projection (VIP) value was generated, indicating the relative contribution of each principal component to OPLS-DA. The significantly different metabolites (SDMs) were considered if they met the *p* < 0.05 (Student’s *t*-test) and VIP > 1 criteria. Finally, the pathways associated with the SDMs were also predicted by MetaboAnalyst and KEGG databases, in which *p* < 0.05 indicated significant enrichment.

#### 2.5.2. Analysis of Enzymes Associated with Energy Metabolism

Data on CMA, T-ATP, and NKA activities were represented as mean ± standard error of the mean (n = 6) and analyzed utilizing the SPSS 26.0 program. Further, the data were assessed using the Shapiro–Wilk normality test and Levene heteroscedasticity test. Finally, one-way ANOVA with Tukey’s post hoc test evaluated the notable variances among the data. Statistically significant differences among the treatments were determined by *p*-values < 0.05.

## 3. Results

### 3.1. Impact of TiO_2_ NPs on the Energy Metabolism Enzymes of Pearl Oysters

**Figure 1:**
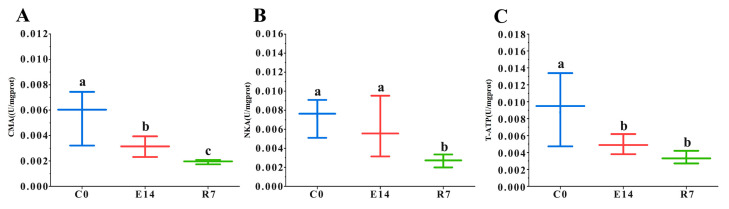
Impact of TiO_2_ NPs on the energy metabolism enzymes of pearl oysters. (**A**) CMA, (**B**) NKA, and (**C**) T-ATP activity levels in the hepatopancreas of *P. f. martensii* among different groups (n = 6). Different letters (a, b and c) mean a significant difference among different groups (*p* < 0.05).

The exposure of pearl oysters to TiO_2_ NPs considerably influenced the activities of energy metabolism-related enzymes (Figure 1). The TiO_2_ NPs exposure significantly decreased CMA and T-ATP activities (*p* < 0.05), and even after the recovery phase (7 days), the CMA and T-ATP activities remained significantly lower than the control without exposure (*p* < 0.05). Meanwhile, no significant difference was observed in NKA activity between E14 and C0 (*p* > 0.05). However, the activity of NKA significantly decreased in the recovery phase compared with the control (*p* < 0.05).

### 3.2. Overall Variations in Metabolites

**Figure 2:**
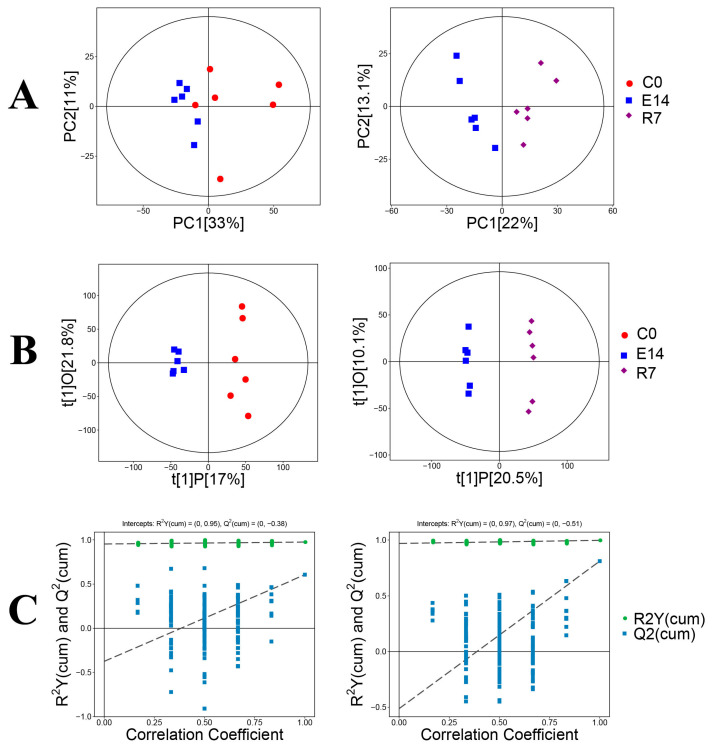
PCA model score scatter plot, OPLS-DA model, and permutation test for C0 and E14, E14 and R7. The PCA model (**A**), OPLS-DA model (**B**), and permutation test of the OPLS-DA model (**C**) were derived from metabolomics profiles.

The study detected 11,812 valid peaks for the pearl oyster samples via LC–MS. The PCA scatter plot displayed groups of comparable data points closely grouped together, while dissimilar data points were more spread out (Figure 2A). The R^2^X value for the PCA was 0.548 between C0 and E14 and 0.564 between E14 and R7. In addition, in the scatter plot, all data points fell within the 95% confidence interval. These findings suggested further analysis of the metabolic datasets.

Therefore, OPLS-DA was performed to investigate the metabolic patterns further and understand the group distinction. Figure 2B shows the OPLS-DA results for pearl oyster gills. In the OPLS-DA scatter plot, all samples were within the 95% confidence interval. In addition, in the OPLS-DA model, the R^2^X, R^2^Y, and Q^2^ scores between C0 and E14 were 0.388, 0.975, and 0.605, respectively, and those between E14 and R7 were 0.306, 0.997, and 0.81, respectively. The R^2^Y and Q^2^ intercepts were 0.95 and −0.38 between C0 and E14 and 0.97 and −0.51 between E14 and R7 (Figure 2C).

### 3.3. Differential Metabolites in Pearl Oysters Exposed to TiO_2_ NPs

**Figure 3:**
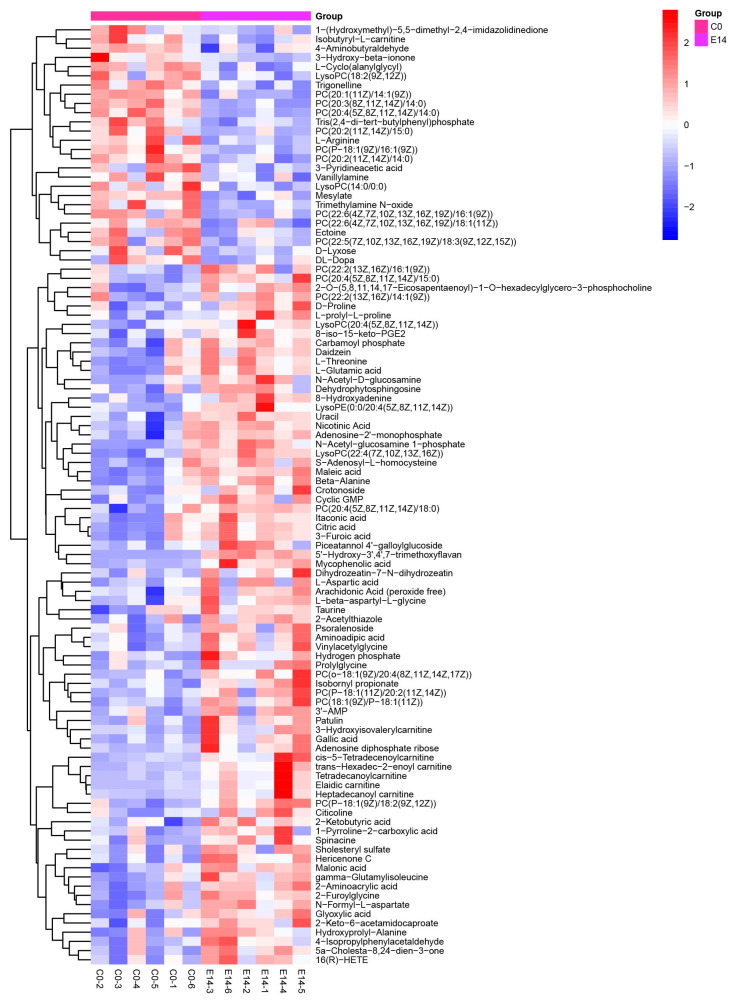
Hierarchical clustering analysis on SDMs between C0 and E14. The relative metabolite level is depicted according to the color scale. Red and blue indicate upregulation and downregulation, respectively.

**Figure 4:**
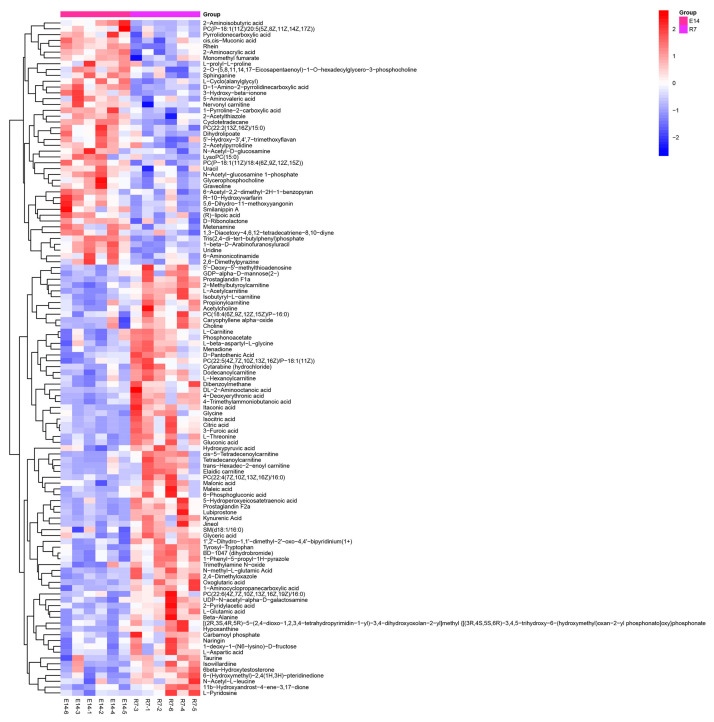
Hierarchical clustering analysis on SDMs between E14 and R7. The relative metabolite level is depicted according to the color scale. Red and blue indicate upregulation and downregulation, respectively.

Further, we screened the SDMs from the identified metabolites in pearl oysters exposed to TiO_2_ NPs. The heatmaps displayed many SDMs in the pearl oysters (Figure 3 and Figure 4). Specifically, 102 SDMs were obtained between groups C0 and E14 and 116 SDMs between groups E14 and R7. Among these, 76 metabolites, including taurine, acetylcholine, citric acid, maleic acid, D-proline, glutamic acid, and itaconic acid, were significantly accumulated in group E14 rather than in group C0. By contrast, 26 metabolites, such as L-Arginine, Isobutyryl-L-carnitine, and trigonelline, were considerably lower in group E14 than in group C0. In addition, 74 metabolites, including acetylcholine, maleic acid, 6-phosphogluconic acid, isocitric acid, glutamic acid, and itaconic acid, were significantly accumulated in group R7 than group E14. By contrast, 42 metabolites, such as uracil, glycerophosphocholine, and N-acetyl-D-glucosamine, were considerably lower in group R7 than in group E14.

### 3.4. Pathway Enrichment Analysis Based on SDMs

**Figure 5:**
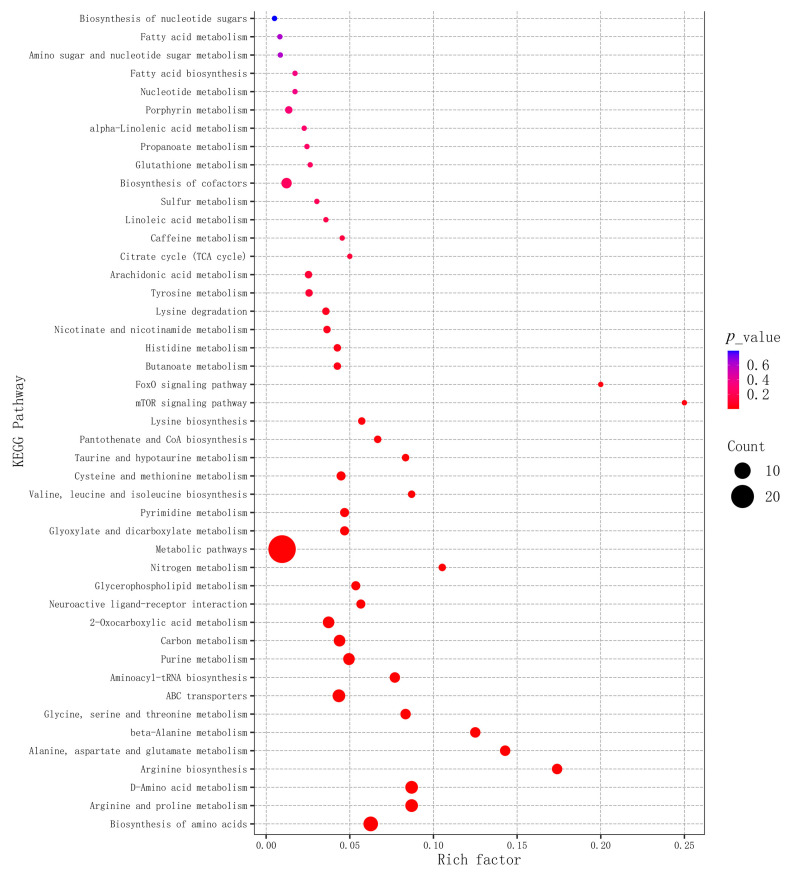
KEGG Enrichment bubble for groups between C0 and E14.

**Figure 6:**
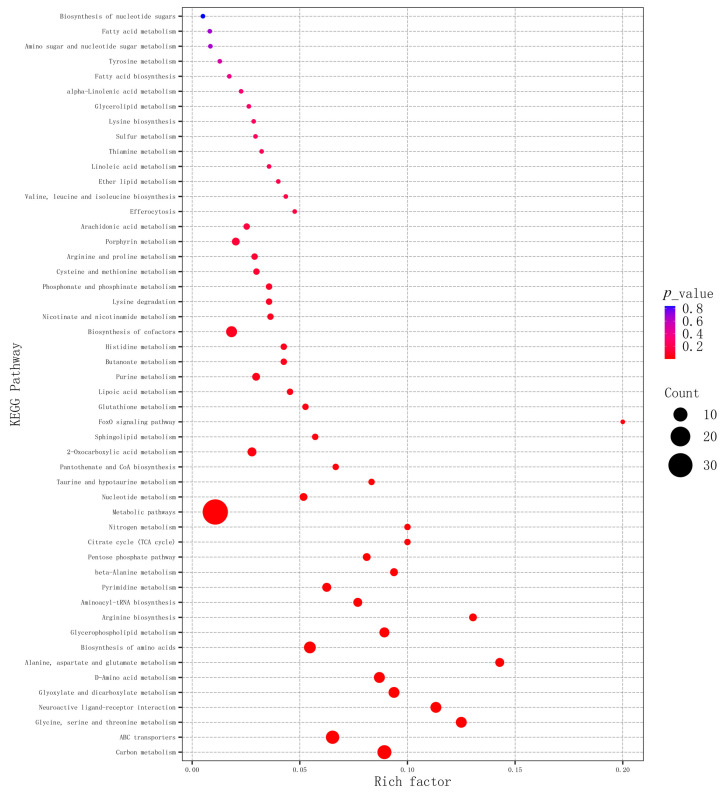
KEGG Enrichment bubble for groups between E14 and R7.

The SDMs were further introduced into MetaboAnalyst to identify the potential metabolic pathways influenced by TiO_2_ NPs. The bubble plots displayed the major pathways associated with the SDMs in the gill tissue (Figure 5 and Figure 6). A total of 28 pathways were identified between C0 and E14 (Figure 5). Such as, Alanine, aspartate, and glutamate metabolism, D-Amino acid metabolism, Neuroactive ligand–receptor interaction, and Taurine and hypotaurine metabolism were enriched between groups C0 and E14. Additionally, 25 metabolic pathways were found between E14 and R7 (Figure 6). Among these, Glutathione metabolism, Carbon metabolism, Glycine, serine and threonine metabolism, and Citrate cycle (TCA cycle) were enriched between E14 and R7.

## 4. Discussion

Changes in environmental conditions induce severe physiological strains and disrupt the intracellular balance in an organism. Specifically, the intertidal bivalves face an array of demanding ecological elements, such as sudden shifts in hypoxia [22], salt levels [37], and temperature [38], alongside exposure to contaminants [39]. Thus, to thrive, these intertidal bivalves have developed highly intricate strategies that assist in modifying their physiology and metabolism and help in adapting to tough environmental circumstances [40]. Furthermore, TiO_2_ NPs in aquatic environments pose a potential threat to aquatic organisms [8]. In this context, the present study reveals the metabolic response mechanism of pearl oysters exposed to TiO_2_ NPs, which provides valuable information for assessing the potential negative effects of TiO_2_ NPs on pearl oysters.

### 4.1. Impact of TiO_2_ NPs on the Oxidative Balance and Immunity

The present study detected a substantial rise in glutamic acid in the gills of pearl oysters after 14 days of TiO_2_ NPs exposure, even with a short recovery phase. This rise in glutamic acid may potentially contribute to oxidative stress within the oysters [21]. Generally, glutathione metabolism manages the reactive oxygen species (ROS) by transforming the reduced glutathione into their oxidized form (GSH into GSSG). This transformation aids in the conversion of electrophilic oxidants like hydrogen peroxide into water molecules [41,42]. Thus, under several physiological and pathological situations, the GSH: GSSG ratio serves as a biomarker of oxidative stress [43]. Glutathione is biosynthesized within the cytosol by utilizing cysteine, glycine, and glutamic acid as the precursors by the action of glutamate–cysteine ligase and glutathione synthetase. Specifically, cystathionine, a product of the trans-sulfuration pathway, supplies cysteine for synthesizing glutathione [42,44]. Thus, despite the absence of changes in glutathione levels, increased levels of glutamic acid indirectly indicate enhanced synthesis of glutathione in response to more ROS production. In agreement with these observations, various studies have emphasized the significance of the glutathione pathway in regulating ROS levels in marine bivalves under pathogenic stress [42,45]. Other researchers have validated the importance of glutamic acid in metabolism and accepted that a higher extracellular accumulation of glutamic acid indicates potential tissue damage [46]. Similar accumulation patterns have been observed in Nile tilapia (*Oreochromis niloticus*) following environmental challenges [47] and the hemolymph of New Zealand clams (*Crassula aequilatera*) during storage conditions [48]. These observations suggest tissue damage due to ROS accumulation, consistent with the activity of oxidative stress-related enzymes previously [34]. Venter et al. [49] report that abalone (*Haliotis midae*) accumulates conjugation of fatty acids with carnitine to detoxify in hypoxic conditions. Moreover, at the E14 stage, isobutyryl-L-carnitine showed a downregulation, while at the R7 stage, it showed an upregulation, indicating that isobutyryl-L-carnitine plays a detoxifying role. Furthermore, in the E14 and R7 samples, we found an increase in L-acetylcarnitine, propionylcarnitine, 2-methylbutyroylcarnitine, L-carnitine, and elaidic carnitine, which can be explained by inhibiting oxygen oxidation and redox imbalance, leading to a rise in related carnitine levels.

Itaconic acid (commonly known as ITA) is a metabolite recognized for its antimicrobial properties, and marine mussels can synthesize ITA to enhance their immune system when combating pathogen infections [50]. Young et al. [45] initially recognized itaconic acid in the Pacific oyster larvae (*Crassostrea gigas*) infected by Ostreid herpesvirus. In addition, New Zealand Green Shell™ mussels (*Perna canaliculus*) infected with Vibrio spp. have elevated levels of itaconic acid detected in various tissues [42]. These reports indicated that ITA potentially serves as a broad-spectrum antimicrobial compound in both vertebrates and invertebrates. In the present study, after 14 days of TiO_2_ NP exposure and 7 days of recovery, the itaconic acid content was higher than that at the start of the treatment. This result indicates that the immune cells of pearl oysters can produce ITA like the bacteria-infected mammalian cells, suggesting ITA may be involved in anti-inflammatory reactions.

### 4.2. Impact of TiO_2_ NPs on Neurotransmitter Signals

Neuroactive ligand–receptor interaction signaling pathway was directly related to neuro function [51]. In the present experiments, we observed that a few SDMs participated in the neuroactive ligand–receptor interaction. The cholinergic system performs a crucial role in regulating innate immunity and inflammation of bivalve mollusks [19]. Acetylcholine is found not only in the central nervous system but throughout the nervous system of invertebrates and it also regulates various animal physiological functions [1]. In transplanted pearl oysters, the cholinergic system controls DNA repair and apoptosis by modulating Ca^2+^, JAK/STAT, NF-kB, and MAPK signaling pathways [52,53]. The acetylcholine binds to α7 nicotinic acetylcholine receptor and inhibits NF-kB and JAK/STAT pathways, reducing the inflammatory response [54]. Additionally, Liu et al. [55] discovered that the acetylcholine-treated oysters lowered the lipopolysaccharide-induced immune response through lower hemocyte phagocytosis and apoptosis rates. Similarly, in the scallops (*Chlamys farreri*), the cholinergic system gets triggered after the immune stimulations and achieves immunomodulation [56]. It has also been reported that the acetylcholine accumulated after transplantation functions through nAChRs by inhibiting the NF-κb pathway in the pearl oyster [57]. Recently, Wu et al. [19] discovered that acetylcholine controls immune and inflammatory reactions during the initial phases of grafting in pearl oysters. This cholinergic neurotransmitter also gets broken down into acetate and choline by acetylcholinesterase. Various ecotoxicological studies in invertebrates have utilized acetylcholinesterase inhibition as a neurotoxicity biomarker [58,59]. Zhang et al. [60] detected a decrease in acetylcholine levels in the adult Manila clams (*Ruditapes philippinarum*) gills after 24 h of exposure to copper at high and low doses. Cappello et al. [61] also noted a drop in acetylcholine levels in mussel gills at a polluted site. However, Guan et al. [16] found increased levels of acetylcholine after exposing *Tegillarca granosa* to TiO_2_ NPs. Similarly, we detected increased levels of acetylcholine in the gills of pearl oysters exposed to TiO_2_ NPs, which may be due to a decrease in the levels of its corresponding regulatory enzyme acetylcholinesterase [16], but further experimental validation is required. These observations thus indicate that the increase in acetylcholine negatively influences the neurotransmission system in pearl oysters exposed to TiO_2_ NPs, modifying the signaling of neurotransmitters. Similarly, the results of our previous experiments showed that TiO_2_ NPs negatively affected the nervous system of pearl oysters [62]. After a short recovery, the level of acetylcholine was still significantly increased, indicating that the neurological effects of TiO_2_ NPs on pearl oysters were sustained, but whether a longer recovery could restore them to normal still needs further study.

### 4.3. Impact of TiO_2_ NPs on Energy Metabolism and Osmotic Regulation Ability

The present study also detected changes in metabolites related to energy metabolism, suggesting that TiO_2_ NPs strongly influence energy pathways. The TCA cycle is a common pathway for the oxidative decomposition of sugars, amino acids, and lipids [38]. We detected elevated concentrations of various metabolites from the tricarboxylic acid (TCA) cycle (citric acid, isocitric acid, and maleic acid) in group R7 compared to the exposed group (E14). Previous studies documented a similar rise in compounds of the TCA cycle in bivalves exposed to marine pathogens [42] or various external pressures [63,64]. Likewise, substantial elevation in fumaric acid, succinic acid, and malic acid was noted in clams stored under normal temperatures in contrast to those stored on ice [50]. These reports suggest environmental stress disrupts the TCA cycle and interferes with energy metabolism. In addition, the hepatopancreas serves as a digestive and metabolic organ in bivalves, and we found that the activities of energy metabolism-related enzymes (CMA, NKA, and T-ATP) were decreased in the hepatopancreatic tissues of pearl oysters after exposure to TiO_2_ NPs, which is consistent with the down-regulation of expression of energy metabolism-related genes (5′-AMP activated protein kinase, Pyruvate kinase, Succinyl-CoA synthetase) hepatopancreatic tissues observed in our earlier study [34]. Therefore, we conclude that TiO_2_ NPs inhibit energy metabolism in gill tissue and hepatopancreatic tissue of pearl oysters. After a brief recovery, we still observed that the contents of citric acid, isocitric acid, and maleic acid were still increased compared to the exposed group (E14), and the activities of CMA, NKA, and T-ATP were still significantly lower than those of the control group, suggesting that the brief recovery did not restore the affected energy metabolism to normal levels.

Our study also detected changes in the concentrations of osmolytes in pearl oysters under exposure to TiO_2_ NPs. Taurine is a critical osmolyte that plays crucial roles in various biological processes; it functions as a cell membrane stabilizer and facilitator in the movement of numerous cations [65]. In addition, small molecules known as organic osmolytes regulate osmotic balance in marine species through different metabolic pathways [66]. In aquatic invertebrates like mollusks, these osmolytes are crucial for cellular-level osmotic adaptation. These compounds accumulate or release within cells and help adjust to changing salinity levels while preserving cell volume [61]. Thus, bivalves exhibit higher levels of organic osmolytes than other metabolites [61]. High levels of organic osmolytes were detected in mussel gills after 30 days in a contaminated environment, suggesting a possible disturbance in osmoregulation [61]. Similar trends were found in the gills of the Manila clam after exposure to benzo (a)pyrene at environmentally relevant concentrations (0.02 and 0.2 μM) [60,67]. Our current research detected substantial upregulation of taurine in pearl oysters exposed to TiO_2_ NPs, which suggests that TiO_2_ NPs interfered with the osmotic regulation.

## 5. Conclusions

The present study unveiled the metabolic response of *P. f. martensii* to TiO_2_ NPs exposure and the crucial metabolites and pathways associated with the response. The TiO_2_ NPs may inhibit the energy metabolism function of gill and hepatopancreas of *P. f. martensii*; meanwhile, TiO_2_ NPs may affect the normal functioning of immune, oxidative stress, and osmoregulatory functions of *P. f. martensii* gill tissues, and even may result in neurotoxicity. These findings also provide a reference for analyzing the biological adaptations of *P. f. martensii* to TiO_2_ NPs stress and the negative impact of TiO_2_ NPs on bivalve shellfish. Future studies should be required for a better assessment of these issues, such as whether a longer recovery period can restore their normal levels.

## Data Availability

Data will be made available upon request.
